# Remnants, LDL, and the Quantification of Lipoprotein-Associated Risk in Atherosclerotic Cardiovascular Disease

**DOI:** 10.1007/s11883-022-00994-z

**Published:** 2022-02-17

**Authors:** Chris J. Packard

**Affiliations:** Institute of Cardiovascular and Medical Sciences, University of Glasgow, Glasgow, G12 8QQ Scotland UK

**Keywords:** Cholesterol, VLDL, Chylomicrons, Non-HDL cholesterol, Apolipoprotein B, Guidelines

## Abstract

**Purpose of Review:**

Implementation of intensive LDL cholesterol (LDL-C) lowering strategies and recognition of the role of triglyceride-rich lipoproteins (TRL) in atherosclerosis has prompted re-evaluation of the suitability of current lipid profile measurements for future clinical practice.

**Recent Findings:**

At low concentrations of LDL-C (< 1.8 mmol/l/70 mg/dl), the Friedewald equation yields estimates with substantial negative bias. New equations provide a more accurate means of calculating LDL-C. Recent reports indicate that the increase in risk per unit increment in TRL/remnant cholesterol may be greater than that of LDL-C. Hence, specific measurement of TRL/remnant cholesterol may be of importance in determining risk. Non-HDL cholesterol and plasma apolipoprotein B have been shown in discordancy analyses to identify individuals at high risk even when LDL-C is low.

**Summary:**

There is a need to adopt updated methods for determining LDL-C and to develop better biomarkers that more accurately reflect the abundance of TRL remnant particles.

## Introduction

Two sets of observations have triggered renewed interest in improving the quantitation of lipid-associated risk in cardiovascular disease. The first was the demonstration in the proprotein convertase subtilisin/kexin 9 (PCSK9) inhibitor trials that achieving very low levels of LDL cholesterol (LDL-C) led to a further decrease in the risk of atherosclerotic cardiovascular disease (ASCVD) [1]. Second, genetic studies have revealed that plasma triglyceride (TG) is a probable causal risk factor for ASCVD [2, 3], bringing to a close (for many commentators at least) decades of controversy concerning the role of TG-rich lipoproteins (TRL)—chylomicrons and VLDL—in atherosclerosis [2, 3, 4•, 5••, 6]. These seminal investigations were followed by consistent reports that plasma TG was a significant contributor to the residual risk of an ASCVD event in patients who had well-treated LDL-C levels [7–10]. Consequently, there is now a twin focus on improving the accuracy and precision of various methods for assessing LDL-C at levels below 1.8 mmol/l (70 mg/dl), and on the development of biomarkers that capture satisfactorily the ASCVD risk associated with elevated plasma TG. In regard to the latter, attention is currently centred on the cholesterol content of TRL and their remnants [2, 3, 4•, 5••, 6].

Revisions to international guidelines for the prevention of cardiovascular disease have incorporated these key observations, and modified their recommendations and treatment strategies accordingly [11•, 12]. There is now a move to more aggressive LDL-C lowering goals; in the case of the latest European guidelines [11•], the goal in the highestrisk category is < 1.4 mmol/l (55 mg/dl), and for those with repeated events within a 2-year period, it is < 1.0 mmol/l (40 mg/dl). As set out below, measuring LDL-C in this range—below 1.8 mmol/l—presents a challenge to many clinical laboratories. Guidelines have also promoted a broader approach to lipid-associated risk assessment. Subsidiary goals have been recommended for non-HDL cholesterol (non-HDL-C) and plasma apolipoprotein B (apoB) since these parameters provide an index of all potentially atherogenic lipoprotein species in the bloodstream [11•]. Cogent arguments have been made for the more widespread adoption of these as biomarkers of risk due to their ease of measurement and possible superior reflection of ASCVD risk in people with elevated plasma TG, especially those who are obese or who have type 2 diabetes [13•, 14••, 15, 16].

The following discussion reviews the merits of recent novel approaches to determining lipoprotein biomarkers in these areas of interest and identifies the challenges that remain.

## LDL – Cholesterol, Particles, and Subfractions

**Fig. 1:**
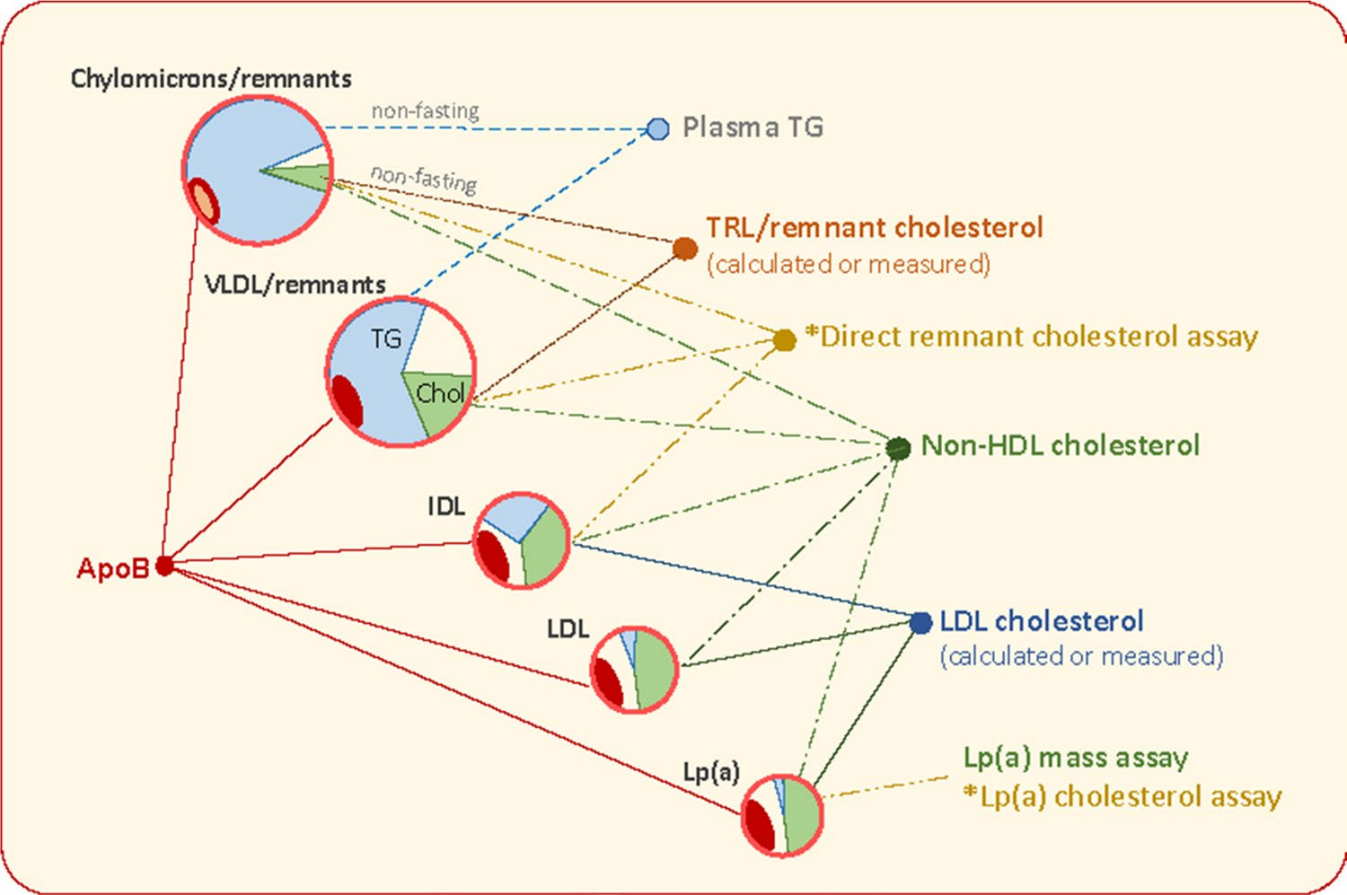
Definitions of biomarkers for lipid-associated risk of atherosclerotic cardiovascular disease. ***Plasma TG*** is mainly transported in VLDL in the fasting state. When non-fasting samples are taken, there is a variable contribution from chylomicrons. ***TRL/remnant cholesterol*** can be measured as the cholesterol content of the d < 1.006 g/ml density fraction isolated from plasma by centrifugation. It includes VLDL and if non-fasting chylomicron cholesterol. There is no clear distinction at present between newly secreted VLDL or chylomicrons and their partially lipolysed products ‘remnants’. These particles are defined together as ‘TRL/remnants’. TRL/remnant cholesterol can be calculated from the plasma TG concentration using the Friedewald, Martin/Hopkins, or Sampson equation [17, 21, 22••]. ***Non-HDL cholesterol*** is the measured content of all apoB-containing lipoproteins, i.e. total cholesterol minus HDL cholesterol. ***ApoB*** is the measured concentration of this apolipoprotein in plasma. It includes apoB100 in all particles and apoB48 in chylomicrons/remnants if they are present. ***LDL cholesterol*** can be measured directly by homogeneous assay, measured after centrifugation/ precipitation by the reference method, or calculated as total cholesterol minus HDL-C minus VLDL-C (= TRL/remnant cholesterol) estimated using Friedewald, Martin/Hopkins, or Sampson equations. Latest developments (*) are a **direct remnant** assay to measure cholesterol in TRL and IDL particles [25•] and a **Lp(a) cholesterol** assay [26•]

Most guidelines from international and national authorities recognise LDL-C as the primary target for prevention of ASCVD in at-risk individuals. It is used to categorise the risk status of patients alongside clinical history and other factors such as elevated blood pressure, presence of type 2 diabetes, and smoking habit [11•, 12]. Furthermore, goals for treatment use achieved LDL-C as the main metric to determine success and to judge the need for more aggressive therapy. LDL-C for decades has been estimated in most clinical chemistry laboratories by a calculation based on the formula derived by Friedewald et al. [17] since the reference method which involves centrifugal separation of the major lipoprotein classes—beta quantification—is expensive, laborious, and time-consuming [18]. Based on the observation that for the majority of the population, a reasonable value for VLDL cholesterol (VLDL-C) can be derived from the plasma TG level (TG divided by 5 on a weight basis); LDL-C was calculated as ‘total cholesterol minus measured HDL-C minus estimated VLDL-C’ (Fig. **1**). However, the systematic shortcomings of this approach became evident from the results of the PCSK9 inhibitor trials where participants recorded very low LDL-C on active drug with median LDL-C about 0.9 mmol/l [1, 19]. LDL-C estimated by the Friedewald formula in subjects on PCSK9 inhibitors showed a marked negative bias compared with centrifugal methods [20]. A second, long-recognised issue with LDL-C as calculated by the Friedewald formula was inaccuracy at elevated plasma TG levels above 4.5 mmol/l (400 mg/dl) [21, 22••, 23, 24]. Again, LDL-C tended to be underestimated and this led to problems in deciding the best treatment options in patients with moderate to severe hypertriglyceridemia.

It was recognised that, if possible, refinement of the formula for calculation of LDL-C was required to allow the continued use of this inexpensive option in assessing goal achievement according to the latest guideline recommendations. The first major revision by Martin et al. [21] varied the factor applied to plasma TG to derive a value for VLDL-C according to the measured non-HDL-C level. When the Martin/Hopkins formula was employed in situations where LDL-C was low such as in the FOURIER trial, the negative bias versus the reference method was largely eliminated [20]. Recently, Sampson et al. [22••] examined further the non-linearity of the association of VLDL-C with plasma TG and generated a more complex but ostensibly more accurate means of estimating LDL-C. This further refinement minimises the bias seen both at low LDL-C levels and in patients with high TG. The potential for misclassification of patients based on LDL-C is diminished when using either the Martin/Hopkins or the Sampson formula and all laboratories should consider implementing these new methods, especially if direct LDL-C assay is not available routinely. A number of evaluations of the various formulae to derive LDL-C have been published recently [23, 24, 27, 28].

A consequence of raised plasma TG is not only the generation of elevated levels of remnant particles (as discussed below) but also perturbation in LDL structure. It was recognised in the 1980s that LDL exhibited size heterogeneity that became increasingly evident as TG rose above 1.5 mmol/l (120 mg/dl). The LDL size profile was altered from a ‘pattern A’—mainly particles of normal diameter—to a ‘pattern B’ in which small, dense particles were predominant [29–31]. Small, dense LDL on the basis of its greater propensity to be oxidised and higher binding potential for arterial wall proteoglycans [31, 32] is considered more atherogenic than its normal-sized counterpart and so may contribute to the increased ASCVD risk seen in hypertriglyceridemia [31]. Measurement of the LDL size profile is possible using a variety of techniques, based on resolving the particle types present in plasma, including gel electrophoresis, centrifugation, and ion-mobility [29, 30, 33]. However, these are regarded as specialised techniques and are not widely available. Other approaches more amenable to routine use include kits for the determination of the cholesterol content in small, dense LDL in plasma [34], and NMR-based methods that can determine the lipoprotein size profile based on deciphering the relative amounts of particles present from the NMR spectra generated by constituent lipid groups (cholesterol, triglyceride, phospholipid) [35, 36]. Increasing awareness of the compositional heterogeneity within the LDL density range has led also to an ongoing debate as to the utility of determining LDL particle numbers rather than LDL-C as the best measure of atherogenic risk [37]. The concept is that it is the particles that are responsible for depositing their contained cholesterol in the artery wall and thereby initiating or promoting growth of atherosclerotic lesions. Particles differ in their cholesterol content with smaller species having a lower cholesterol content than larger ones, and yet having a higher atherogenic potential [31]. On this basis, it is argued that, in general, particle numbers (or apoB as discussed below) provide a superior index of risk [13•, 35–37], and there may be value in determining specifically the plasma concentration of small, dense LDL. Indeed, epidemiological studies have reported that this lipoprotein sub-species is an independent risk factor for ASCVD [38•, 39]. In an additional adaption of the method of calculating lipoprotein levels from the basic plasma lipid profile, Sampson et al. recently derived an equation for the estimation of the concentration of small, dense LDL cholesterol [40]. If it can be established as a useful marker of risk, then estimation of its abundance using such an equation or by direct measurement may be a useful addition to lipid profiling in at-risk individuals.

When measuring LDL-C at low levels—especially < 1.8 mmol/l—a further complication is that the contribution from lipoprotein (a) (Lp(a)) can become significant (Fig. **1**) [41]. This lipoprotein comprises an LDL particle complexed via a disulphide bond to apolipoprotein (a), a protein of highly variable length produced in the liver. The cholesterol within Lp(a) is in the LDL component and accounts for about 30% of the mass [42, 26•]. In many people, the plasma Lp(a) concentration is low and the associated cholesterol a minor portion of LDL-C. However, Lp(a) levels vary widely and in some at-risk individuals can be a substantial fraction of the measured LDL-C [42]. Now that treatment strategies are focussed on achieving very low LDL-C, the assessment of Lp(a) has become an issue and novel methods have been developed to measure Lp(a)-cholesterol and so improve the accuracy of LDL-C estimation in these circumstances [26•]. A revised perspective of LDL-C-associated risk thresholds corrected for Lp(a)-cholesterol has been devised [43].

## TRL/Remnant Cholesterol—Definitions and Measurement

There have been a number of recent excellent reviews on the role of TRL in atherosclerosis, including the consensus statement from the European Atherosclerosis Society (EAS) [2, 3, 4•, 5••, 44–46]. There appears to be general agreement that findings from genetic studies, clinical trials, and epidemiology have provided strong evidence to support the causal association of TRL with atherogenesis. The original concern that this relationship was attributable to confounding factors, especially the inverse link between raised TG and low HDL-C [4•, 44], has been allayed and TG is emerging as an important risk factor and possible drug target. However, as highlighted in the EAS consensus publication, it is not clear which aspect of human TG biology is responsible for promoting the development of arterial wall lesions; is it the TG present in TRL, products of TG hydrolysis such as partial glycerides or fatty acids, the cholesterol contained in all TRL particles, or the population of remodelled ‘remnant’ particles formed by the action of lipoprotein lipase on newly secreted chylomicrons and VLDL [5••, 8, 45–47]. By analogy with LDL, most attention is being paid at present to ‘remnant cholesterol’ as the best index of ASCVD risk linked to plasma TG [2, 4•, 5••, 6, 45–50]. Remnants are enriched with cholesteryl ester and cholesterol compared to their parent (i.e. newly secreted) lipoproteins, and TRL/remnant cholesterol has been shown in a number of reports to be an independent risk factor for ASCVD in populations [2, 5••, 6, 7, 8, 48–50]. It has also been found to predict atheroma progression and risk of a future ASCVD event—so-called residual risk—in subjects in clinical trials where LDL-C has been lowered substantially (near to recommended goals) [7, 8, 15, 50].

In the absence of a formal definition of a remnant and clarity as to how best to measure its concentration in the bloodstream, care must be taken in interpreting the findings of these association studies [5••, 47, 52]. In the majority of reports, remnant cholesterol is counted as the total cholesterol found in TRL e.g. [7, 9, 48–52]. However, others have included VLDL cholesterol plus the cholesterol in intermediate density lipoprotein (IDL) [25•]. Where non-fasting samples are used, there is the potential for measured TRL/remnant cholesterol to include contributions from both VLDL (apolipoproteinB100 containing lipoproteins of liver origin) and chylomicrons (apoB48 containing lipoproteins of intestinal origin) [5••, 52, 53]. Expert opinion indicates that such a non-fasting assessment may be at least as informative as fasting measurements [52, 53]. Arguably, in our present state of knowledge, the inclusive term ‘*TRL/remnant cholesterol*’ should be adopted to define the cholesterol content of this class of lipoproteins. This usage accepts that currently, we cannot separate newly secreted particles and their contained cholesterol from partially lipolyzed cholesterol-enriched remnants (Fig. 1). It is likely that TRL/remnant cholesterol is proportionate to the concentration of remnants present in the d < 1.006 g/ml density range even if it is not an accurate measure of remnant abundance.

Current approaches to assessing TRL/remnant cholesterol are based on calculation from the plasma lipid profile, analysis of NMR spectra, immunologically based assays, and techniques based on selective assay by particle size. In a pooled study of asymptomatic cohorts over 18 years of observation, Quipse et al. found that a calculated ‘remnant cholesterol’ (in the following discussion, the terminology employed by the various authors is provided in quotation marks) derived using the Martin/Hopkins equation (approximately equal to VLDL (TRL) cholesterol) predicted ASCVD events independent of LDL-C and apoB [50]. The main approach they used was a discordancy analysis of groups with high remnant-C and low LDL-C versus other combinations of these variables. It was found that compared to a reference group of low remnant-C (< 24 mg/dl) plus low LDL-C (< 130 mg/dl), those with high remnant-C plus low LDL-C had a 65% greater risk (hazard ratio of 1.65 [CI 1.45–1.89]) for ASCVD. These results are in broad agreement with earlier findings from Danish cohorts [2, 6, 25•] that examined the relationship of non-fasting ‘remnant-C’ to ischaemic heart disease risk. Based on both genetically determined and measured lipid levels, Varbo et al. reported that a 1.0 mmol/l change in remnant-C was associated with greater risk of IHD than the same increment in LDL-C [6]. Studies that looked specifically at subjects on statin treatment have also concluded that elevated TRL/remnant-C is an independent risk factor. Vallejo-Vaz et al. [7] examined the association of ‘TRL-C’ with cardiovascular event rates in the well-known Treat-To-New Targets trial, a test of high-dose versus low-dose atorvastatin. TRL-C was calculated as the difference between non-HDL-C and LDL-C (estimated by Friedewald or by centrifugation where TG was > 400 mg/dl). Higher levels of TRL-C were associated with higher event rates in the low-dose statin arm of the trial and the benefits of higher-dose statin therapy were more evident in those with the highest TRL-C at baseline, and linked to the changes in both TRL-C and LDL-C. These findings taken together support the recommendations that TRL/remnant-C should be considered in assessing risk prior to intensification of lipid-lowering therapy [11•].

Since lipolysis of the bulk of the TG which resides in the interior of spherical large TRL results in a substantial reduction in particle size, VLDL remnants almost by definition lie in the size range of small VLDL and IDL [5••]. Techniques that can determine lipoprotein concentration across the size spectrum can therefore be used to assess the abundance of remnant particles [54]. Classical techniques such as size-exclusion chromatography [55] and ultracentrifugation [5••, 54, 55] have been employed to measure and characterise remnants but these are not suited for large-scale studies. NMR spectroscopy of plasma provides an indication of lipoprotein concentration by size [36, 37, 56, 57]. Using the NMR-based Nightingale analytical platform, Balling et al. [57] reported approximately one-third of non-fasting total plasma cholesterol resided in the ‘remnant’ lipoprotein fraction defined as subclasses of VLDL plus IDL, and that remnants and LDL particles carried comparable amounts of cholesterol in the circulation. On the basis of these measurements, Balling and co-workers then proceeded to examine what proportion of the overall ASCVD risk in the Copenhagen population could be attributed to VLDL-C versus LDL-C. They found that one-half of overall risk was accounted for by variation in VLDL-C [58], and, furthermore, that the hazard ratios for myocardial infarction per unit change in VLDL-C and IDL-C exceeded that for LDL-C. These findings are provocative and somewhat at odds with the quantitation of VLDL + IDL cholesterol by classical centrifugal methods (as recognised by the authors) where the observed cholesterol content of these fractions is around 15 to 20% of the total [59]. The results of the NMR quantitation in the study by Balling et al. [58] were the subject of correspondence in the journal [59, 60]. All writers agreed that the definition of a remnant and the inclusion of IDL cholesterol with LDL-C (as in beta quantification) or with VLDL-C can lead to confusion. Moreover, results from NMR-based techniques for lipoprotein quantitation are highly dependent of the computational method used to extract size and concentration data from the NMR spectra. It is important therefore as pointed out by the correspondents that even in large-scale epidemiological studies, attention is paid to traceability of the assay results to well-defined reference methods [59]. Further work needs to be undertaken in this area ahead of any widespread adoption of such techniques in clinical practice.

A recently developed, alternative technique for quantifying ‘remnant’ cholesterol was tested in a study in which calculated and directly measured remnant cholesterol were compared [25•]. Calculated remnant-C was defined as total minus HDL-C minus LDL-C and measured remnant-C as the results obtained from a two-step assay where LDL- and HDL-sized particles are degraded and the cholesterol content of the remaining lipoproteins determined. Both measures were related to risk of ASCVD in the general Danish population but use of directly measured remnant-C was found to identify a proportion of at-risk individuals, about 5% of the population, that were not classed as at increased risk on the basis of the calculated value.

In an alternative approach to investigating TRL remnants ‘remnant-like particles’ (RLP) are isolated using antibodies that capture and remove lipoproteins containing apoA1 (HDL) and apoB with the protein conformation found in LDL [61]. The assay is conducted on plasma and the unbound lipoprotein fraction comprising both apoB48-containing particles (chylomicron remnants) and apoB100-containing particles (those that do not express epitopes for the antibody used) is quantified by measuring its cholesterol content. RLP-C is correlated strongly with other measures of TRL/remnant-C and with plasma TG and is associated with increased ASCVD risk [62–64]. The technique has also been employed to separate and characterise the lipoproteins present in the RLP fraction [55]. These include chylomicron remnants, VLDL subfractions, and IDL-sized particles. These findings emphasise the heterogeneous nature of remnant lipoproteins as a class of particles, and the fact that the species present will depend on the degree of hypertriglyceridemia [5••, 45, 46, 47, 53]. That is, individuals with elevated plasma TG not only have higher levels of remnants but the size and composition of these particles differ from those found in subjects with low plasma TG [5••].

## Non-HDL Cholesterol and ApoB as Integrated Biomarkers

Recognition that all apoB-containing lipoproteins are atherogenic and the diminished importance of HDL as a potential target for intervention has led to renewal of the debate as to the utility of employing a readily and reliably measured, single biomarker of lipid-associated risk—non-HDL cholesterol and/or plasma apoB. The assay of an analyte that encompasses LDL, TRL/remnants, and Lp(a) (Fig. 1), all of which have strong genetic evidence to support their causal relationship to ASCVD [2, 3, 4•, 5••, 64, 65], is attractive and guidelines provide subsidiary goals for these biomarkers [11•] (a fuller assessment of the role of Lp(a) in ASCVD and the measurement issues involved in quantifying accurately this lipoprotein species has been the subject of recent reviews and innovations [26•, 42, 52, 66, 67]). A further argument in favour of their adoption is that this would circumvent problems (as recounted above) concerning the definition of remnants and whether or not to include IDL with VLDL/chylomicrons in the remnant lipoprotein category [13•, 68]. The caveat, of course, is that employing a single variable such as non-HDL-C to predict ASCVD risk and as a guide to treatment success does not allow for the possibility that its component parts have varying strengths of association with risk. For example, if a given increment in TRL/remnant-C carries a twofold greater risk than the same increase in LDL-C [25•, 46, 50, 58], then for many individuals at-risk, especially those with hypertriglyceridemia or type 2 diabetes, a non-HDL-C-based risk prediction derived from population statistics driven mainly by variation in LDL-C will be an underestimate.

The potential utility of non-HDL-C as an integrated biomarker was explored in a pooled analysis of 44 cohorts from 19 countries across Europe, Australia, and North America [14••]. Non-HDL-C was related to cardiovascular disease outcomes over a median observation period of 13.5 years in over 500,000 subjects free of (overt) cardiovascular disease at baseline. Thirty-year risk of an ASCVD event increased stepwise from < 7% for non-HDL-C < 2.6 mmol/l to 33.7% for non-HDL-C ≥ 5.7 mmol/l in both men and women. The data were incorporated into a tool that predicted risk and the clinical benefits of reducing non-HDL-C. This study provides an example of the potential usefulness of a simple biomarker in predicting long-term risk of ASCVD. Non-HDL-C as a predictor of residual risk in statin-treated subjects was examined in a discordancy analysis of 13,015 individuals on statins in the Copenhagen General Population Study [69••]. The cohort was divided into subgroups with LDL-C or non-HDL-C above or below the median value in the population. It was observed that compared to the reference group of below median LDL-C and non-HDL-C, those with an above median non-HDL-C but below median LDL-C had a significantly increased risk of myocardial infarction (hazard ratio 1.78 [CI 1.35–2.34]) and total mortality. This type of evidence supports the concept that a broader measure of atherogenic lipoproteins than LDL-C may be a better guide as to the need for further intervention, for example with add-on therapy such as ezetimibe or a PCSK9 inhibitor.

ApoB is the other candidate biomarker that reflects the total concentration of atherogenic lipoproteins in the circulation. Each of LDL, remnants, and Lp(a) contains only one apoB peptide per particle and so the amount of apoB is a direct measure of particle number (Fig. 1). Sniderman [13•] has argued that given the known heterogeneity in the cholesterol content of particle in the VLDL-LDL spectrum, only apoB provides an unambiguous index of particle number. It is also recognised that it is the apoB-containing particle that is the vehicle for delivery of cholesterol to the artery wall [31]. The study by Johannesen et al. [69••] examined also the predictive power of apoB in statin-treated subjects in a discordancy analysis, and as for non-HDL-C found apoB to be superior to LDL-C as a predictor of future ASCVD risk. Both apoB and non-HDL-C appeared equally good at predicting risk of a myocardial infarction but apoB was the more important risk factor of the two when considering total mortality. There are further arguments supporting the widespread adoption of apoB as a primary biomarker of ASCVD risk and treatment guide including its ease of measurement and standardisation across assays [13•, 70]. However, there is still a clear steer by guideline writers to use LDL-C as the main target since it is the key metric on which the body of clinical trial evidence is founded [71].

It may be that we have to await the results of further trials before contemplating a move to non-HDL-C or apoB as a single measure of lipid-associated risk. The upcoming study of pemafibrate in hypertriglyceridemic patients [72] will help quantify the benefits of TG lowering as will future outcome trials of agents directed towards apolipoprotein CIII and angiopoietin like 3 protein [73]. Only when there is clear clinical trial evidence that a specific reduction in TRL/remnant-C has the same per mmol/l benefit as a drop in LDL-C will there be a solid scientific basis to use non-HDL-C or apoB as a guide to risk in all subjects and a suitable goal on which to base therapeutic decisions. The assumption has been that this is the case [13•] but recent data, as set out above, has questioned this conclusion.

## Conclusions

As we move to an era of aggressive LDL lowering using more expensive agents, it is important to employ measures of lipid-associated ASCVD risk that are fit-for-purpose. LDL-C is still the primary focus of international expert guidelines both for assessing risk and setting treatment goals. The shortcomings of the traditional Friedewald calculation based on a standard laboratory lipid profile are now well understood. Superior equations have been published that address issues of incorrect reporting and patient misclassification especially at low LDL-C levels and in patients with hypertriglyceridemia. All clinical chemistry laboratories should implement the new methods of estimating LDL-C as an aid to judging if and when to proceed to combination lipid-lowering therapy. The current interest in treating individuals with a high residual risk due to elevated plasma TG is directing attention to the atherogenic potential of remnant lipoproteins and how best to measure their abundance in the circulation. More work needs to be done to characterise these lipoprotein particles and if possible develop a lipidomic or proteomic signature that would allow their unambiguous assay. In the meantime, use of TRL/remnant cholesterol serves as a surrogate biomarker. As recommended in the ESC/EAS guidelines, the prudent approach is to employ LDL-C as the primary goal and non-HDL-C or apoB as an ancillary target. As the recent discordancy analyses suggest once the LDL-C goal has been achieved, careful attention should be paid to the risk linked with elevated levels of other apoB-containing lipoproteins as reflected in non-HDL-C or apoB.
